# Pfam: the protein families database

**DOI:** 10.1093/nar/gkt1223

**Published:** 2013-11-27

**Authors:** Robert D. Finn, Alex Bateman, Jody Clements, Penelope Coggill, Ruth Y. Eberhardt, Sean R. Eddy, Andreas Heger, Kirstie Hetherington, Liisa Holm, Jaina Mistry, Erik L. L. Sonnhammer, John Tate, Marco Punta

**Affiliations:** ^1^HHMI Janelia Farm Research Campus, 19700 Helix Drive, Ashburn, VA 20147 USA, ^2^European Molecular Biology Laboratory, European Bioinformatics Institute (EMBL-EBI), Wellcome Trust Genome Campus, Hinxton, Cambridge CB10 1SD, UK, ^3^Wellcome Trust Sanger Institute, Wellcome Trust Genome Campus, Hinxton, Cambridge CB10 1SA, UK, ^4^MRC Functional Genomics Unit, Department of Physiology, Anatomy and Genetics, University of Oxford, Oxford, OX1 3QX, UK, ^5^Institute of Biotechnology and Department of Biological and Environmental Sciences, University of Helsinki, PO Box 56 (Viikinkaari 5), 00014 Helsinki, Finland and ^6^Stockholm Bioinformatics Center, Swedish eScience Research Center, Department of Biochemistry and Biophysics, Science for Life Laboratory, Stockholm University, PO Box 1031, SE-17121 Solna, Sweden

## Abstract

Pfam, available via servers in the UK (http://pfam.sanger.ac.uk/) and the USA (http://pfam.janelia.org/), is a widely used database of protein families, containing 14 831 manually curated entries in the current release, version 27.0. Since the last update article 2 years ago, we have generated 1182 new families and maintained sequence coverage of the UniProt Knowledgebase (UniProtKB) at nearly 80%, despite a 50% increase in the size of the underlying sequence database. Since our 2012 article describing Pfam, we have also undertaken a comprehensive review of the features that are provided by Pfam over and above the basic family data. For each feature, we determined the relevance, computational burden, usage statistics and the functionality of the feature in a website context. As a consequence of this review, we have removed some features, enhanced others and developed new ones to meet the changing demands of computational biology. Here, we describe the changes to Pfam content. Notably, we now provide family alignments based on four different representative proteome sequence data sets and a new interactive DNA search interface. We also discuss the mapping between Pfam and known 3D structures.

## INTRODUCTION

Pfam is a database of curated protein families, each of which is defined by two alignments and a profile hidden Markov model (HMM). Profile HMMs are probabilistic models used for the statistical inference of homology (1,2) built from an aligned set of curator-defined family-representative sequences. A high-quality seed alignment is essential, as it provides the basis for the position-specific amino-acid frequencies, gap and length parameters in the profile HMM. In Pfam, the profile HMM is searched against a large sequence collection, based on UniProt Knowledgebase (UniProtKB) (3), to find all instances of the family. Sequence regions that score above the curated threshold that is set for each family to eliminate false positives (the so-called gathering threshold) are aligned to the profile HMM to produce the full alignment. Curated entries are referred to as Pfam-A entries. The profile HMMs are built and searched using the HMMER software suite (http://hmmer.janelia.org) (4,5).

Sometimes, a single profile HMM cannot detect all homologues of a diverse superfamily, so multiple entries may be built to represent different sequence families in the superfamily. Such related Pfam-A entries are grouped into clans (6). In an effort to be comprehensive, automatically generated entries, called Pfam-B, are built from sequence clusters not currently covered by Pfam-A entries.

Pfam data are available in a variety of formats, which include flatfiles (derived from the MySQL database) and relational table dumps, both of which can be downloaded from the FTP site (ftp://ftp.sanger.ac.uk/pub/databases/Pfam). The Pfam website (available at http://pfam.sanger.ac.uk/and http://pfam.janelia.org/) provides different ways to access the database content, providing both graphical representations of and interactive access to the data.

In the 2012 article (7), much of the content was focused on curation details. In this article, we focus on describing the new and updated data features provided in the database and by the website. Besides adding new features, it is also important to indicate those that are no longer available, many of which have been removed due to our drive to scale with the growing influx of new sequences.

## PFAM STATISTICS

The current release of Pfam, version 27.0, contains 14 831 Pfam-A families. Of these families, 4563 have been classified into 515 clans. Compared with Pfam 26.0, there has been an increase of 1159 families (1182 new entries have been added and 22 entries have been removed) and 16 new clans, with an additional 320 families having been classified into clans. The Pfam-A families in release 27.0 match 79.9% of the 23.2 million sequences and 58% of the 7.6 billion residues in the underlying sequence database. This corresponds to a negligible percentage increase in sequence and residue coverage (<0.5%), but reflects a significant amount of curation effort. These statistics mask the fact that the underlying sequence database has increased by 7.3 million sequences, a number greater than the entire sequence database of Pfam 23.0, which contained 5.3 million sequences.

Two of the main sources for generating the new families added to release 27.0 were Protein Data Bank (PDB) structures (8) and human sequences. We have made a concerted effort to build families from CATH domains (http://www.cathdb.info/) (9) that did not match a Pfam family in Pfam 26.0. To do so, we used jackhmmer, a program within the HMMER3 software that allows a sequence to be iteratively searched against a sequence database. One hundred new Pfam-A families were built using the sequence of a CATH domain to initiate a jackhmmer search against our underlying sequence database (three iterations were run using an E-value threshold of 0.001). Our curators then used the output from the last iteration of the jackhmmer program as the basis for generating the seed alignment of a new Pfam-A entry. We have also built families for *Homo sapiens* sequences that did not have a match in Pfam 26.0. By taking the Swiss-Prot collection of human sequences (∼20 000 sequences) and excluding those sequences matched by a Pfam-A entry, each remaining sequence was used to initiate a jackhmmer search. Again, Pfam-A entries were built from the jackhmmer output. By building families in this way, we have increased the sequence coverage of the Swiss-Prot set of human sequences by almost 5% and the residue coverage by 2.2%. The Pfam 27.0 sequence coverage of Swiss-Prot human sequences is now 90.5% and the residue coverage is 45.1%. We will continue to work on incorporating more human regions into Pfam-A, as there is still much to be gained at the residue level. However, attaining high residue coverage in human is complicated by the large fraction of intrinsic disorder found in the regions that are not currently covered by Pfam-A families [discussed further in (10)]. In addition to using CATH domains and human sequences as starting points for new Pfam families, we continue to add families built from Pfam-B entries, as well as from community submissions received via our helpdesk. We have received 135 direct submissions from our seven registered external contributors, who have our database curation tools installed locally to facilitate automated deposition.

In 2012, we described the introduction of Wikipedia as a platform for community-based functional annotation (7). Since release 26.0, the first to include links to Wikipedia articles, we have tried to link as many Pfam-A families as possible to those articles that best describe their biology. The number of families linking to a Wikipedia article increased from 4942 in 26.0 to 5663 families in release 27.0, an increase of 721. Of these 721 new links, 391 were added to old families and 330 were added to new families in Pfam 27.0. Some articles may be linked to many Pfam-A families, but the number of unique Wikipedia articles also rose by 311, from 1016 in 26.0 to 1327 in 27.0. As described previously, we operate a manual approval system that allows us to view all changes to our linked articles. Although the number of newly linked articles has increased, we have also observed a steady stream of edits to many of the linked articles. Most edits are simple format or typographic improvements, but many have also provided valuable scientific content, including significant improvements to and expansion of important articles. For example the Wikipedia article on EGF-like domains was significantly expanded in October 2012.

## RECENT CHANGES TO THE DATABASE CONTENT

### Removing dubious sequences from the underlying database

Each Pfam release is calculated against a fixed sequence database, called pfamseq, which is derived from UniProtKB (3). At the beginning of a release cycle, we take a copy of the current version of UniProtKB and process it in two ways, the second of which is a novel addition for release 27.0. First, we remove sequences that contain non-consecutive regions. The linear sequence-information in these proteins will be inaccurate, as adjacent residues in the sequence can flank an intervening number of unsequenced residues. There are currently <1000 UniProt entries that contain non-consecutive sequence regions. The second, new processing step is the removal of sequences derived from spurious open reading frames, which are identified by searching AntiFam (11) models against the sequence database. In release 27.0, the models from AntiFam version 2.0 identified 2829 sequences for removal.

### Family full alignments and trees

When building a Pfam release, we aim to ensure that the same set of post-processing operations are performed on all families regardless of size, thereby providing consistency both to the database and to the website. One of the distinguishing features of Pfam compared with most other protein family databases is our provision of full alignments. Unsurprisingly, however, with the exponential growth of the underlying sequence database, we have observed a similar dramatic increase in the size of our full alignments. Although generation of these alignments does not currently present a scalability problem, aiding human interpretation through visualization has become increasingly difficult. Most approaches for facilitating alignment visualization natively in the browser do not scale well. Applets, such as the Jalview alignment viewer (12), partly solve the problem, but require Java to be installed and coupled to the browser.

For example, the largest Pfam-A family (version 27.0) with >363 000 matches to the profile HMM is the ABC transporters family (ABC_tran, accession PF00005)—its full alignment is thus too large to be useful for most purposes. The seed alignment, by contrast, contains just 55 representative sequences, which may be an insufficient number to represent the sequence diversity within the family. To provide more useable samples of the sequence diversity within a family, we now calculate model-matches for four additional sequence sets, based on ‘Representative Proteomes’ (RPs) (13). For the ABC_tran family, the RP alignments range in size from approximately a quarter of the size of the full alignment to less than one tenth.

In an RP set, each member proteome is selected from a grouping of similar proteomes. The selected proteome is chosen to best represent the set of grouped proteomes in terms of both sequence and annotation information. The grouping of proteomes is based on a clustering of UniProt, UniRef50, and includes all complete proteome sequences. In each cluster, sequences have ≥50% identity and have at least an 80% overlap with the longest sequence. The similarity of two proteomes is determined by considering just the clusters containing sequences from either of the two proteomes. The two proteomes are grouped when the fraction of clusters that contain sequences from both proteomes out of the subset of proteome-specific clusters exceeds a given threshold. This threshold is termed the co-membership threshold. The percentage threshold of co-membership (or common clusters) can be adjusted down to produce larger groupings, and hence less redundant sequence sets.

We use the RP sequence sets constructed using co-membership thresholds of 75, 55, 35 and 15%, giving a range of sequence redundancy for each family. Using representative proteomes has the advantage that it still allows for organism-specific copy numbers to be assessed, a feature that can be lost when using global non-redundancy thresholds on an entire sequence database. However, the major advantage for Pfam is the dramatic reduction in the size of the family full alignments, as shown in Table 1, which illustrates the reductions with increasingly redundant RPs for the 10 biggest families in Pfam. The RP sets do not currently include viruses, and so for some families such as GP120, there may not be a match to the RP sets.

**Table 1.** The reduction in size of RP versus full alignments

Family identifier (accession)	Seed	Full	RP75	RP55	RP35	RP15
ABC_tran (PF00005)	55	363 409	26% (93 265)	21% (77 150)	16% (57 358)	8% (28 903)
COX1 (PF00115)	94	254 351	1% (2006)	0.7% (1661)	0.4% (1218)	0.2% (538)
zf-H2C2_2 (PF13465)	163	227 898	61% (138 033)	27% (60 664)	15% (34 039)	9% (21 562)
WD40 (PF00400)	1804	193 252	65% (125 805)	52% (100 531)	36% (69 386)	23% (21 562)
MFS_1 (PF07690)	195	181 668	30% (55 719)	25% (55 719)	17% (55 719)	8% (55 719)
RVT_1 (PF00078)	152	172 360	5% (8257)	4% (6662)	3% (5373)	2% (3604)
BPD_transp_1 (PF00528)	81	156 339	23% (36 523)	19% (29 422)	14% (22 134)	7% (10 630)
Response_reg (PF00072)	57	151 337	29% (44 329)	25% (37 848)	20% (29 453)	10% (15 208)
GP120 (PF00516)	24	146 453	N/A	N/A	N/A	N/A
HATPase_c (PF02518)	659	129 386	28% (36 085)	24% (30 935)	19% (24 121)	10% (12 473)

The seed alignment is used to construct the profile HMM and contains a representative set of sequences of the family. The full alignment contains all hits in pfamseq scoring above the gathering threshold. In Pfam 27.0, we have introduced four additional alignments based on RPs, which contain decreasing amounts of sequence redundancy from RP75 to RP15. For each RP data set, the percentage reduction in the size of the full alignment is shown, with the number of sequences given in brackets.

The reduction in the size of the full alignments varies from family to family, reflecting in part the bias in the sequence database. Overall, across the whole of the database, using RP at 75, 55, 35 and 15% co-membership thresholds results in average alignment sizes that are, respectively, 38.8, 29.7, 20.4 and 11.6% of the full alignment size. As the number of sequences in the sequence database increases, we anticipate that the alignments based on RPs will grow at a more linear rate and provide a more convenient way of sampling the full alignment sequence diversity.

As illustrated in Table 1, the full alignment size for the top 10 families ranges from 129 000 to 363 000 sequences. With alignments of this size, it is no longer practical to calculate the neighbour-joining trees provided in previous Pfam releases. Before release 27.0, these approximate neighbour-joining phylogenetic trees (with bootstrapping values based on 100 replicas) were used to order the alignments, such that phylogenetically related sequences would be grouped together. From release 27.0 onwards, the full alignments are ordered according to the HMMER bit score of the match, with the highest scoring sequence found at the top of the alignment. The same phylogenetic trees are still provided for the seed alignments, but are merely a guide as they are calculated with the FastTree approximation algorithm (14). The seed alignment sequences remain ordered according to the calculated tree.

**Figure 1.**
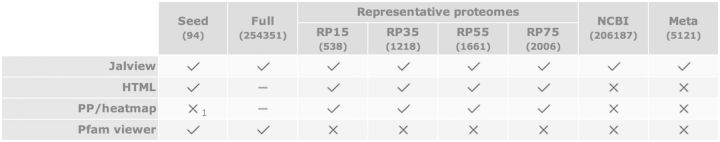
Table from the ‘Alignments’ tab of the family page for COX1 (PF00115), showing the availability of different views and different alignments for COX1. The posterior probability-based alignment is only available for the full alignments as it is derived from the alignment of a sequence to the HMM, as indicated by the subscript 1 in the corresponding seed alignment cell.

In the Pfam website, we use two different colouring schemes when displaying our alignments in a web browser: the Clustal scheme (15), based on the chemical properties of the amino acids found in the column, and a heat-map scheme that reflects the posterior probability of alignment confidence (16). However, the complexity of the large multiple sequence alignments, in terms of gaps and variation, can result in vast numbers of HTML elements being generated to mark up an entire alignment. The maximum number of elements that can be displayed depends on the user’s browser and hardware, but, in an effort to protect users from attempting to view alignments that are unlikely ever to be rendered, we only make HTML versions of alignments that contain 5000 sequences or fewer. In an effort to convey which options for viewing an alignment are available for a given family via the website, we present a table indicating the availability of the alignment view option (Figure 1).

## SEARCH-INTERFACE DEVELOPMENTS

As the volume of data in Pfam increases, it is important to make that data even more discoverable. Before Pfam 27.0, keyword searches were performed via the backend MySQL database, using the ‘fulltext’ indexing method offered by the database engine. However, the performance of this search was deteriorating as the database grew with each release, particularly when queried with common words. To ensure future scalability, keyword searches are performed outside of the database, using Apache Lucy (http://lucy.apache.org), a tool specifically designed for full-text indexing. This has allowed us to tailor the searches to improve specificity (any query term of ≥2 characters will be used as a query), such that all query-matching strings, including substrings, are found for text associated with a Pfam-A family, structures and ontology; the sequence-annotations are also indexed, but, due to the quantity of text, this index is built only to match complete words. Results from the different text indexes are amalgamated and ordered, based on the index—prioritized in the following order: Pfam, sequence annotation, structure, Gene Ontology and InterPro—and the query term score. Keyword searches are now interactive, typically returning in <100 ms.

### Faster interactive DNA searches

**Figure 2.**
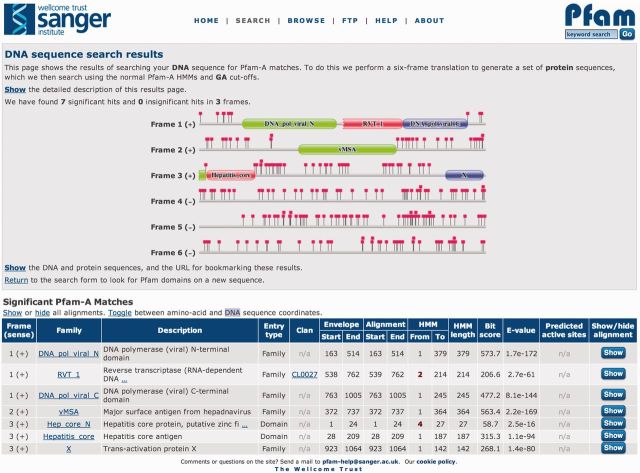
Results from searching Pfam with the Hepatitis B virus isolate G376-7, complete genome (GenBank accession AF384371.1), providing a striking example of overlapping genes. The six reading frames are displayed graphically in the top box of the results page. All three reading frames from the positive strand contain matches to Pfam-A, which are tabulated below. The positions of stop codons are indicated by the square lollipops. The results are shown with the ‘protein’ coordinates of the open reading frame, but it is also possible to toggle this to DNA sequence coordinates. This search tool accepts sequences up to 80 000 nucleotides in length, and searches the Pfam-A HMM library using the gathering threshold.

Pfam has provided an asynchronous DNA search tool since 2000 (17). The function of this tool is to try to identify the presence of Pfam-A families on an input DNA sequence, with results emailed to the user. Currently, it is not possible to compare directly a protein profile HMM against a DNA sequence using HMMER3. The previously described search was constructed around the GeneWise software (18), and would compare the DNA sequence to the protein profile HMMs via a gene model. The GeneWise software was originally written for profile HMMs built using the HMMER2 software suite, and although it is possible to back-convert HMMER3 models to HMMER2 format, we found that there was a significant loss in sensitivity for these searches. HMMER3 models tend to have lower relative entropy per position due to the altered prior weighting, compared with HMMER2. This, coupled with the tuning of GeneWise specificity, could account for the loss of sensitivity. However, the increased speed of HMMER3 presented an alternative approach for the detection of Pfam matches on DNA sequences. As opposed to the more sophisticated gene structure-aware approach used previously, we now can perform a standard six-frame translation on the DNA, and search each of the resulting ‘protein’ sequences against the Pfam-A library. This brute-force approach with HMMER3 is sufficiently quick to allow the use of the same interface as we use for the interactive protein sequence searches, thus unifying the sequence search interface for both protein and DNA. In the DNA search results page (Figure 2), each open reading frame is represented graphically, with the positions of the stop codons in the reading frame highlighted by red square lollipops and the positions of any domains represented using the standard Pfam domain representations. The DNA search functionality has also been incorporated into pfam_scan.pl, our downloadable tool for performing sequence searches against Pfam.

### Changes associated with alternative target sequence databases

To streamline the production of the database, we no longer store the matches to the NCBI NR (non-redundant) protein sequence database (19) or our metagenomics sequence collection. We still provide Stockholm formatted alignments of all matches to each family found in these two sequence databases as well as retrieval of sequences via accession (e.g. 22125853 or EBH56784.1). However, the data for non-UniProt sequence pages come from an on-the-fly search of the sequence against the Pfam-A HMM library. Generating the data in this manner not only reduces the time required to populate the database, but also provides a more coherent view of the Pfam match data: overlapping matches arising from other clan families can be removed (previously all matches were reported for the NR and metagenomics sets) using the same rules that are used for UniProtKB sequences. As a result, the view is identical to the UniProt sequence page, where the data are retrieved from the database.

## DEPRECATED FEATURES

In our 2004 article (20), we described the introduction of contextual domain-hits, which used language-modeling techniques to identify weak domain hits that fell just below the gathering threshold but had support from surrounding domains (or contextual information) (21). Unfortunately, the third-party software used to generate such matches in Pfam is no longer supported and the existing implementation fails to scale, both in terms of time and memory, when presented with the tens of millions of matches now reported by Pfam. Although there is merit in providing additional functional annotations via contextual domain-hits, the improved sensitivity offered by HMMER3, the introduction of clans (which allows us to build multiple models for ubiquitous domains that cannot readily be matched by a single model) and/or simply improved models, means that many of these contextual domains are now reported by standard Pfam-A matches (Table 2). Since the last time it was calculated, in 2007, 37% of the previously identified contextual hits (10 559) are now covered by Pfam entries. The majority of contextual hits were for Pfam-A entries of type ‘Repeat’ and the highest proportion of unidentified hits belong to this entry type. This reflects the difficulty we have in generating profile HMMs that are able to detect all instances of a short degenerate, repeating sequence motif. Table 2 summarizes the breakdown of context hits that are now matched in Pfam 27.0.

**Table 2.** Breakdown of contextual hits that are reported by Pfam entries in Pfam 27.0, according to the protein family type

Entry type	% Context regions reported in Pfam 27.0	% Context regions not reported in Pfam 27.0
Family	4	7
Domain	13	13
Motif	<1	2
Repeat	20	41
All	37	63

The percentage reported for each entry type is the fraction out of all of the 10 559 contextual domains, with the total for all domains shown at the bottom of the table.

In addition to removing features based on scalability issues, we also routinely analyze the web server access logs, to assess how the site is used. From such analyses, we have identified that the functional similarity search, which used a similarity tool (22) to identify sets of related Pfam-A families based on functional annotation (Gene Ontology terms), was not being used. We have removed this search facility from the site.

## IMPROVING ACCESS TO PROTEOME DATA

Before release 27.0, Pfam proteome data came from Integr8, a project that has now closed and whose data have been distributed to other EBI resources. We now obtain our complete proteome data directly from UniProt, at the beginning of the release cycle when the sequence database is retrieved. This has resulted in better consistency between the sequence sets, with 40% (9 423 167 sequences) of the 23 193 494 sequences in pfamseq belonging to a complete proteome. Over the past few years, we have received an increasing demand for proteome-centric Pfam data. The data-interface to the proteome data is an area of future development but, to satisfy one of our most common user queries, we now provide a list of all Pfam-A matches per proteome on our FTP site (ftp://ftp.sanger.ac.uk/pub/databases/Pfam/current_release/proteomes). Each list can also be accessed from the corresponding proteome's ‘domain composition’ tab on the proteome-pages in the website.

## REPRESENTING INTRINSIC SEQUENCE DISORDER

**Figure 3.**
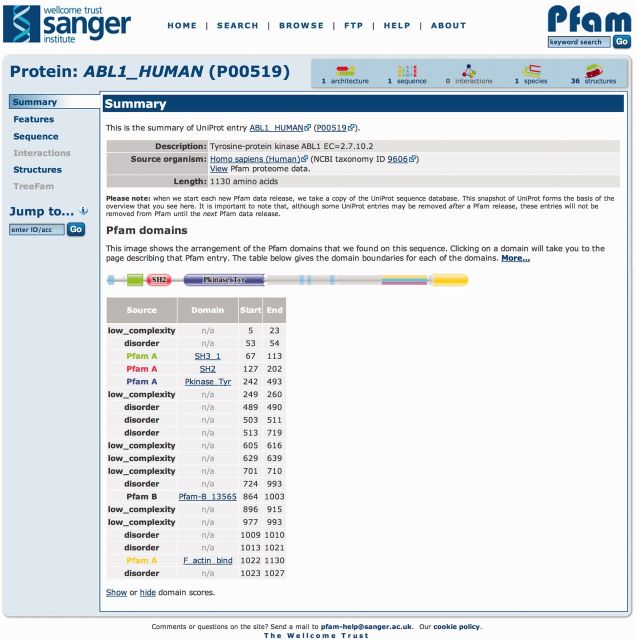
Graphical representation of the Pfam sequence annotations for human tyrosine-protein kinase ABL1 sequence (UniProtKB accession P00519). This sequence matches four different Pfam-A entries, SH3_1 (PF00018), SH2 (PF00017), Pkinase_Tyr (PF007714) and F_actin-bind (PF08919). Between the Pkinase_Tyr and F_actin_bind families is a long region of disorder, indicated by the presence of the grey boxes on the sequence. A disorder prediction does not necessarily mean that the sequence is not conserved, highlighted by the presence of an overlapping Pfam-B region (striped box).

Pfam often quotes ‘sequence coverage’ and ‘residue coverage’ as statistics for tracking the extent of annotation provided by the database. We have previously noted that achieving 100% residue coverage is an unrealistic goal, as every residue in a sequence does not form part of a conserved globular domain (23), such as signal peptides and domain linker regions (short regions are essential for interdomain interactions, folding and stability) (24–27). To aid in the identification of non-globular domain regions, we have displayed the predictions of signal peptides (28), low complexity (29) and coiled-coils (http://www.russelllab.org/cgi-bin/coils/coils-svr.pl) for many years. As part of recent, focused curation efforts aimed at increasing the Pfam-A coverage of the human proteome (10), it became apparent that many regions not covered by Pfam-A are predicted to be intrinsically disordered. Disorder is not an indicator of a lack of function; on the contrary, it has been shown to be involved in cell signaling, protein interactions and regulation (30–33). Some disordered regions are conserved and are found within existing domains, e.g. in PF03250 (Tropomodulin), but they generally appear to be less conserved and/or shorter than globular domains (10), making them more elusive to modeling in a conventional Pfam-A entry. Therefore, to provide a means of identifying more disordered regions in Pfam, we have incorporated IUPred predictions (34,35) (using the long disorder prediction option) for all pfamseq sequences. These data are stored in the MySQL database, and displayed graphically as grey boxes on the website graphical representation of a sequence, as in Figure 3. The IUPred disorder predictions supplement those already produced by SEG (29), which predict a single class of disorder. Although more common to eukaryotes, disordered regions are widespread in UniProtKB. In Pfam 27.0, there were 5.5 million IUPred disorder regions of 50 amino acids or more in length, corresponding to 5.6% of the 7.6 billion sequence residues in the database.

## MAPPING PFAM-A ENTRIES TO PROTEIN STRUCTURES

A recurring issue, and one which is often raised in the literature (36) and by Pfam users, is the mapping of Pfam-A entries to PDB entries, a process that can provide 3D structural information for a protein family. This may seem like a trivial task, whereby one simply extracts all of the protein chains in all of the PDB entries and searches them against Pfam-A. However, although this approach works in principle, in practice it results in many omissions from the mapping. PDB entries frequently include only part of a sequence and the visible fragments are often simply too short to have matches to Pfam profile HMMs that are significant. For example, the crystal structure of the murine class I major histocompatibility antigen H-2D(B) has been determined in complex with a nine amino acid peptide derived from the LCMV gp33 protein (PDB identifier 1S7W) (37). Searching just the gp33 fragment against the Pfam-A models finds no hits. However, by using the residue mapping between PDB structures and UniProtKB entries provided by the SIFTS resource (38), we find that the fragment comes from a larger sequence, UniProtKB accession P07399, in a region that matches the Arena_glycoprot family (Pfam accession PF00798). This demonstrates the importance of using a comprehensive and accurate structure-to-sequence mapping, such as SIFTS, to unify structural and sequence information.

The caveat to the approach described earlier in the text is that structure, mapping and sequence data, from PDB, SIFTS and Pfam, respectively, must be time-synchronized. All resource providers are aware of the issues generated by multiple release cycles and our pipeline has been modified to ensure that, at the point of data acquisition, PDB, SIFTS and UniProt are as tightly synchronized as possible. However, as there is a steady flow of structures into the PDB every week and, since our data are often downloaded and frozen months before a release, it will almost always appear out of date. During the lifetime of a Pfam release, the disparity will become increasingly wide. One solution would be to pull this data in dynamically during a Pfam release, but we are opposed to this approach because we believe that the data in a given Pfam release should be fixed, to provide a stable data source for the community to cite. Should obtaining the latest Pfam-PDB annotation-mapping be paramount, both PDBe (39) and RCSB (40) offer tab-delimited files with the latest mappings (ftp://ftp.ebi.ac.uk/pub/databases/msd/sifts/flatfiles/csv/pdb_chain_pfam.csv.gz or http://www.rcsb.org/pdb/rest/hmmer?file=hmmer_pdb_all.txt). A better solution might be to make more frequent Pfam releases, thereby minimizing the data synchronization lags. Continued improvements in our release pipeline are designed to facilitate shorter release cycles in the future.

## CONCLUSIONS

The core aim of Pfam is to produce protein families that reliably classify as much of sequence space as possible. The database continues to grow and evolve during 2013, with efforts concentrated on adding new families and improving existing ones, while also trying to make the core family data as accessible as possible. The growing sequence database is competing with this effort. We continue to focus attention on meeting the needs of our users, which are often highlighted by recurring user requests. Part of this effort is to identify and remove features that have not been useful to users. It is always tempting to add progressively more features to the database, but this would make it impossible to keep Pfam maintainable in the long term. However, we still encourage the Pfam user community to ask for data sets that are either not provided or not easily accessible. We are committed to producing more frequent releases, a process which may result in further changes to the database and website.

## FUNDING

Howard Hughes Medical Institute Janelia Farm Research Campus (to R.D.F., J.C. and S.R.E); the European Molecular Biology Laboratory, European Bioinformatics Institute (EMBL-EBI) (to A.B. and J.M.); Wellcome Trust [WT077044/Z/05/Z to P.C., R.Y.E., K.H., J.T. and M.P.]. Funding for open access charge: HHMI Janelia Farm Research Campus.

*Conflict of interest statement*. None declared.
